# The complete chloroplast genome of *Agapanthus praecox* (Agapanthaceae), an ornamental and medicinal plant

**DOI:** 10.1080/23802359.2021.1995516

**Published:** 2021-11-03

**Authors:** Yan Dong, Yangyang Zhang, Yan Zhang, Jianhua Yue

**Affiliations:** a Xinyang Agriculture and Forestry University, Xinyang, PR China; b Huazhong Agricultural University, Wuhan, PR China

**Keywords:** *Agapanthus praecox*, chloroplast genome, phylogenetic analysis, Agapanthaceae

## Abstract

*Agapanthus praecox* is a monocotyledonous, herbaceous, and perennial plant, which has been used as an ornamental and medicinal plant. Here, we assembled and characterized the complete chloroplast (cp) genome of *A. praecox* by *de novo* high throughput sequencing. The results revealed that the cp genome of *A. praecox* was 157,038 bp in total length, including a large single-copy (LSC) region of 85,195 bp, a small single-copy (SSC) region of 18,113 bp, and two invert repeats (IR) regions of 26,865 bp. The total plastid genome of *A. praecox* included 132 genes comprising 86 protein-coding genes, 38 tRNA genes, and eight rRNA genes. The phylogenetic analysis was conducted based on the complete cp genomes of 17 species and it indicated that *A. praecox* is closely related to *A. coddi* in Agapanthaceae family.

*Agapanthus praecox* is a monocotyledonous, herbaceous, and perennial plant (Zhang et al. 2010). This species is well-known for its esthetic qualities which include cut flowers, potted plants, floral border plants in the landscape (Zhang et al. 2010). Besides, *A*. *praecox* contains medicinal compounds such as saponins, sapogenins, and phytoecdysteroids (Mori et al. 2014). APG I classification system (1998) placed the genus *Agapanthus* in the family of Agapanthaceae, then Agapanthaceae family has been subjected to Alliaceae family and Amaryllidaceae family in APG II (2003) and APG III (2009) classification system, respectively. To date, APG IV (2016) supported APG III that Agapanthaceae was included in the expanded Amaryllidaceae family. However, there has been considerable debate over the systematic position of the genus *Agapanthus*. In the past few decades, the genus *Agapanthus* has been placed in Liliaceae family, Amaryllidaceae family, and Alliaceae family in different classification systems of angiosperm (Zhang et al. 2010). The chloroplasts (cp) genome has a maternal inheritance and conserved structure, that has been used to examine the developmental and phylogenetic relationships of plants (Wang et al. 2018). Therefore, here we assembled the complete cp genome of *A*. *praecox* based on Illumina pair-end sequencing data and analyzed the characteristic of its cp genome. The results of this study will provide more information for the phylogenetic reconstruction of the genus *Agapanthus*.

*A*. *praecox* leaves specimens were sampled from Henan Province, China (Xinyang Agriculture and Forestry University: 114° 13′ E, 32° 17′ N) and instantly preserved in liquid nitrogen. Later these specimens (YJH01) were stored in the −80 °C refrigerator of Horticultural Plant Biotechnology Laboratory, Xinyang Agriculture and Forestry University. After the DNA extraction from leaf tissues, its quantification was validated by using 1% (*w*/*v*) agarose gel electrophoresis and NanoDrop spectrophotometer 2000. Qualified DNA was used for library construction and sequencing on the Illumina High-throughput sequencing platform (HiSeq2500). Approximately 12.2 GB of raw data were generated with 150 bp paired-end read lengths. The data were filtered by the script in the NOVOPlasty (Dierckxsens et al. 2016). The complete plastid genome of *Agapanthus coddii* (GeneBank accession: KX790363) was chosen as a reference. The plastid genome was assembled by GetOrganelle, it can get the plastid-like reads, and the reads were viewed and edited by Bandage (Wick et al. 2015). The cp genome annotation was assembled based on the comparison by Geneious v 11.1.5 (Biomatters Ltd, Auckland, New Zealand) (Kearse et al. 2012).

The complete cp genome sequence of *A*. *praecox* has been deposited to GenBank under the accession number: MW829770. Raw reads were deposited in the GenBank Sequence Read Archive (SRA: SRX10335872). The complete cp genome of *A*. *praecox* was a circular shape of 157,038 bp in length, with 41.97% GC content. The total plastid genome consisted of four distinct regions, such as a large single-copy (LSC) region of 85,195 bp, a small single-copy (SSC) region of 18,113 bp, and two inverted repeats (IR) regions of 26,865 bp. The complete cp genome consisted of 132 genes, including 86 protein coding genes, 38 tRNA genes, and eight rRNA genes. Phylogenetic analyses including *A. praecox*, *A. coddii*, eight Amaryllidaceae species, four Alliaceae species, and three Liliaceae species, were performed using complete cp genomes. All of the genomes were downloaded from NCBI GenBank. The sequences were aligned by MAFFT v7.307 (Katoh and Standley 2013), and the phylogenetic tree was constructed by MEGA X (Kumar et al. 2018). The robustness of the topology was estimated using 1000 bootstrap replicates with the neighbor-joining method (Tamura et al. 2004). The phylogenetic tree revealed that *A. praecox* is closely related to *A. coddi* in Agapanthaceae family (Figure 1).

**Figure 1.**
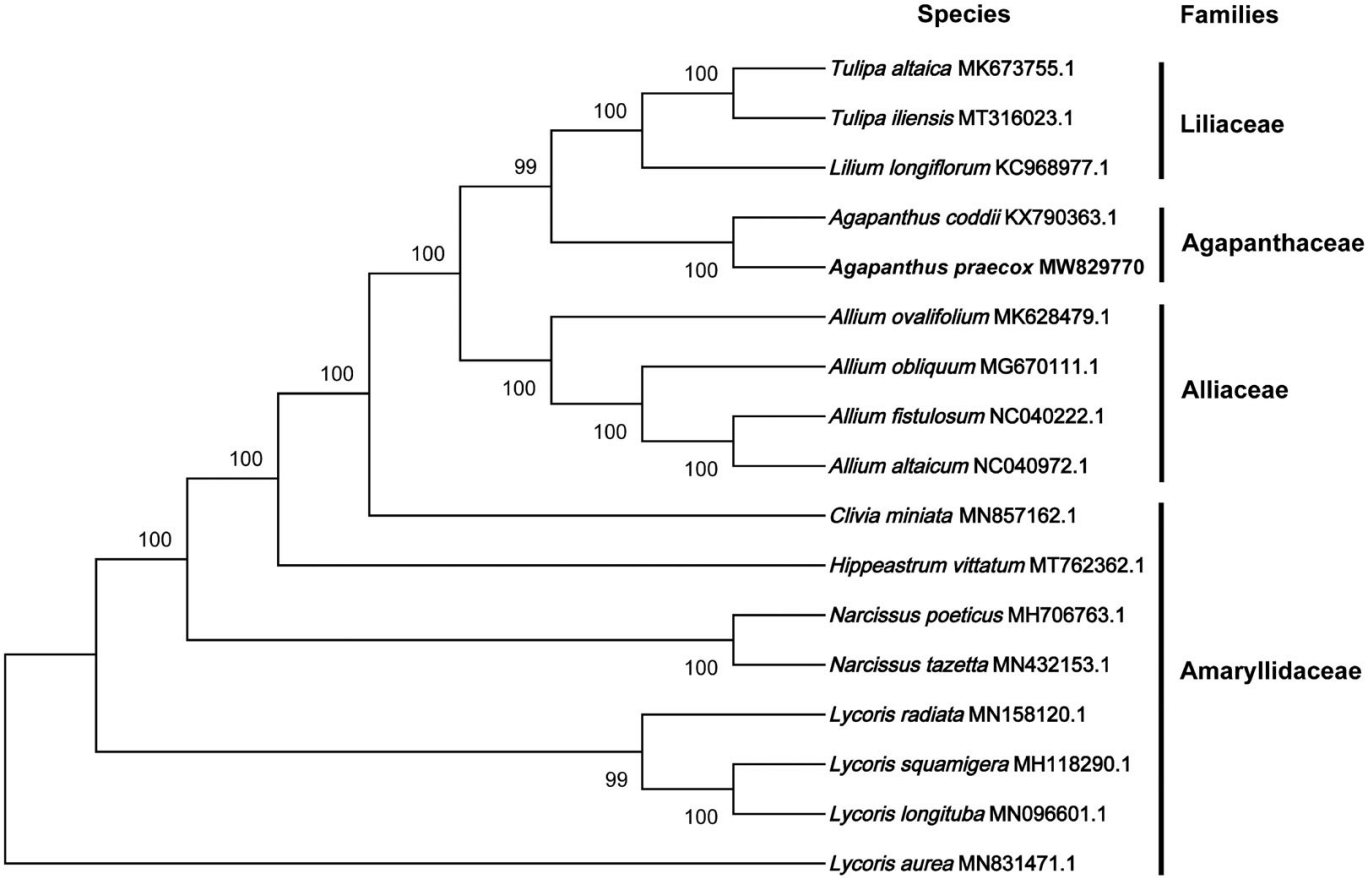
Phylogenetic analysis based on the complete cp genomes. The bootstrap values were shown on the nodes, the species and GenBank accession number were shown at the end of each branch.

## Data Availability

The data that support the findings of this study are openly available at https://www.ncbi.nlm.nih.gov/. The complete chloroplast genome has been deposited in GeneBank with accession number MW829770. And the associated Bioproject, SRA, Bio-sample numbers are PRJNA714137, SRX10335872 and SAMN18290660 respectively.
